# Pleasant touch moderates the subjective but not objective aspects of body perception

**DOI:** 10.3389/fnbeh.2013.00207

**Published:** 2013-12-23

**Authors:** Donna M. Lloyd, Victoria Gillis, Elizabeth Lewis, Martin J. Farrell, India Morrison

**Affiliations:** ^1^Institute of Psychological Sciences, University of LeedsLeeds, UK; ^2^School of Psychological Sciences, University of ManchesterManchester, UK; ^3^Department of Clinical Neurophysiology, Sahlgrenska University HospitalGothenburg, Sweden; ^4^Institute of Neuroscience and Physiology, University of GothenburgGothenburg, Sweden; ^5^Department of Cognitive Neuroscience and Philosophy, University of SkövdeSkövde, Sweden

**Keywords:** CT afferents, insula, pleasant touch, proprioceptive drift, rubber hand illusion

## Abstract

Un-myelinated C tactile afferents (CT afferents) are a key finding in affective touch. These fibers, which activate in response to a caress-like touch to hairy skin (CT afferents are not found in palm skin), may have more in common with interoceptive systems encoding body ownership, than afferent systems processing other tactile stimuli. We tested whether subjective embodiment of a rubber hand (measured through questionnaire items) was increased when tactile stimulation was applied to the back of the hand at a rate optimal for CT afferents (3 cm/s) vs. stimulation of glabrous skin (on the palm of the hand) or at a non-optimal rate (30 cm/s), which should not activate these fibers. We also collected ratings of tactile pleasantness and a measure of perceived limb position, proprioceptive drift, which is mediated by different mechanisms of multisensory integration than those responsible for feelings of ownership. The results of a multiple regression analysis revealed that proprioceptive drift was a significant predictor of subjective strength of the illusion when tactile stimuli were applied to the back of the hand, regardless of stroking speed. This relationship was modified by pleasantness, with higher ratings when stimulation was applied to the back of the hand at the slower vs. faster stroking speed. Pleasantness was also a unique predictor of illusion strength when fast stroking was applied to the palm of the hand. However, there were no conditions under which pleasantness was a significant predictor of drift. Since the illusion was demonstrated at a non-optimal stroking speed an integrative role for CT afferents within the illusion cannot be fully supported. Pleasant touch, however, does moderate the subjective aspects of the rubber hand illusion, which under certain tactile conditions may interact with proprioceptive information about the body or have a unique influence on subjective body perception.

## Introduction

Illusions of body ownership provide a valuable tool by which to probe the mechanisms mediating embodiment. The Rubber Hand Illusion (RHI; Botvinick and Cohen, 1998) in particular is now considered a well-established paradigm by which to manipulate self-representation. When a seen rubber hand is stroked in precise spatial and temporal synchrony with a participant's concealed hand, the majority of people will report perceiving the touch as if coming from the rubber hand, such that the rubber hand *feels* like part of their own body, which may indicate “ownership” (i.e., incorporation into the perceptual body schema) of the rubber hand.

This subjective experience of the illusion is most often measured using standard self-report measures (i.e., Botvinick and Cohen, 1998). These measures are designed to capture three main elements of the illusion: (1) “visual capture” of the proprioceptive position sense of the hand; (2) “body ownership” or the degree to which the rubber hand is perceived to have been incorporated into one's own body schema; and (3) the degree of causal attribution of the illusory touch sensation by touch to the rubber hand. Taken together, these elements provide an index of the strength of the illusion. These subjective reports have been objectively quantified using proprioceptive distortion of the position of the person's real hand, which is felt to be closer to the rubber hand after stroking (usually described as “proprioceptive drift”) and neural activity related to the illusion from functional imaging studies. For example, using Positron Emission Tomography, Tsakiris et al. (2007a) measured the extent of perceptual drift (as a proxy measure of body ownership) after a period of synchronous visuo-tactile stimulation during the RHI. They found that the size of perceptual drift related to the extent of activity in right posterior insula and right frontal operculum of the brain. These results suggest that intersensory matching between visual and tactile events is sufficient for body ownership, when measured as a change in body position sense.

Many investigators believe that the feeling of ownership over the rubber hand occurs because vision of the hand co-occurs with touch of the hand being stroked, and that this distorts proprioceptive information so as to form a meaningful, but illusory, percept. Visual, tactile and proprioceptive signals relating to limb position are integrated in ventral premotor and parietal cortices in humans and non-human primates (Graziano, 1999; Rizzolatti et al., 2002; Lloyd et al., 2003) and previous studies have shown that the degree of premotor activity elicited during the RHI is linearly related to the subjective strength of the illusion (Ehrsson et al., 2004).

However, many people also subjectively report the rubber hand as “feeling like it's my hand” and although visuo-tactile stimulation is a necessary condition for such subjective embodiment of the hand to occur it may not be sufficient. There is now evidence to suggest that the RHI requires more than the involvement of multisensory areas and that the “natural” feeling of body ownership necessitates engagement of emotional systems, which may in turn lead to a stronger illusory experience (Ehrsson et al., 2007). Although multisensory integration between external visual and tactile signals seems a necessary condition for the experience of the illusion, the “feeling” of body ownership is a fundamental aspect of self-awareness and there are now several lines of evidence supporting a role for interoceptive systems in mediating the subjective aspects of this illusion (for a review see Seth, 2013).

Interoception is the encoding and representation of internal bodily signals (i.e., thermal, nociceptive and visceromotor) reporting the body's physiological state (Craig, 2002). Some interoceptive channels also convey motivationally-salient information about the functional state of the body (including environmental and biological threats to bodily integrity), whilst others carry signals that evoke appetitive and positive hedonic responses (e.g., pleasant taste, satiety, and pleasant touch). The subjective experience of the RHI leads to changes in many interoceptive systems including temperature (Moseley et al., 2008), cortical (Lloyd et al., 2006) and skin conductance responses associated with the emotion of fear or threat (Armel and Ramachandran, 2003; Ehrsson et al., 2007), limb-specific temperature decreases and increased intradermal histamine reactivity (Moseley et al., 2008; Barnsley et al., 2011) and even pain processing (Capelari et al., 2009). These findings suggest that artificial limbs can evoke the full range of feelings associated with real limbs and that the physiological state of the owned artificial limb is subject to the same monitoring by the brain's emotional system as the real limb.

The un-myelinated C tactile afferents (CT afferents) that sub-serve pain and temperature sensations also sub-serve pleasant touch (for a recent review see Olausson et al., 2010). CT afferents are slow-conducting, un-myelinated low-threshold mechanoreceptive nerve fibers that carry signals from the receptive fields in the epidermis of mammalian hairy skin (Vallbo et al., 1999; Olausson et al., 2002; Wessberg et al., 2003). They are particularly receptive when a light touch is applied slowly over the skin surface at the optimal stroking speed of 3 cm/s (Löken et al., 2009). It has been proposed that CT afferents play a role in the RHI. For example, a recent study by Crucianelli et al. (2013) found slow velocity touch was perceived as more pleasant and produced higher levels of subjective embodiment during the RHI compared with fast touch. Moreover, this effect applied irrespective of whether the seen hand was a rubber hand or a confederate's hand. However, this study only applied tactile stroking to hairy skin. A similar study by Van Stralen et al. (2012) found similar subjective embodiment as well as a stronger temperature drop and a larger proprioceptive drift after affective touch when compared to regular stroking. These findings provide support for the idea that affective touch, and more generally interoception, may have a unique contribution to the sense of body ownership (although see Schütz-Bosbach et al., 2009 for no change in subjective body ownership when applying pleasant vs. unpleasant tactile materials to either hand during the RHI).

To gain a more complete picture of the role of the interoceptive system in body representation, the current study behaviorally manipulated the activity of these nerve fibers in order to determine whether the perceived pleasantness of visuo-tactile stimulation influenced the occurrence of the illusion and also whether the presence of the illusion could modulate the perceived sensory quality of the tactile stimulus by increasing pleasantness ratings. Higher feelings of pleasantness should accompany the slower of the two stroking speeds (3 cm/s) used in the experiment compared to the faster speed (30 cm/s) and only be present for stimulation to the back of the hand (hairy skin) and not the palm of the hand (glabrous skin). Participants were asked to rate the subjective pleasantness of the touch experienced in each condition so that we could determine the relationship between perceived pleasantness, illusion strength, and stroking speed and location. We also collected an objective measure of perceived limb position: proprioceptive drift. As the CT afferent pathway associated with dynamic affective touch differs anatomically and functionally from that of the myelinated Aβ afferent pathway associated with the rapid sensory processing of touch then the mechanisms responsible for feelings of ownership should differ from those responsible for limb position sense. If pleasant touch predicts the subjective strength of the illusion but has no influence on perceptual drift, this would support a role for interoception in the subjective but not objective aspects of body perception.

## Materials and methods

### Participants

Twenty-four female participants (mean age 19 years) were recruited from the University of Manchester undergraduate student population and provided written informed consent to take part in the study, which had local ethics committee approval. Since high degrees of left-handedness have been seen to correlate with a negative effect on the illusion (Niebauer et al., 2002), the sample was limited to right-handed participants only.

### Design

A 2 × 2 within-participants design was used with the independent variables of site of stimulation (back of hand vs. palm of hand) and speed of stimulation (3 vs. 30 cm/s) and the dependent variables of subjective strength of the illusion (measured from questionnaire items; see Apparatus and Materials), proprioceptive drift (final position—baseline position in cm) and pleasantness of stimulation. The experiment, therefore, had four conditions (the order in which each condition was presented was fully counter-balanced across participants): Condition A (back of the hand stimulated at a speed of 3 cm/s); Condition B (back of the hand stimulated at a speed of 30 cm/s); Condition C (palm of the hand stimulated at a speed of 3 cm/s) and Condition D (palm of the hand stimulated at a speed of 30 cm/s).

### Apparatus and materials

Participants sat at a table across from the experimenter and placed their right hand inside a specially constructed black wooden box (dimensions: 40 cm wide × 27 cm deep × 12 cm high). The index finger of the right hand was placed on a pre-determined spot concealed from view. The side of the box facing the experimenter was open and through it, the experimenter could stroke the participant's hand using their index finger. A small wooden block, painted black, was placed on top of the box to obscure the participant's hand from view and to prevent the participant from viewing the experimenter stroking the hand. To enable participants to make proprioceptive judgments about the location of their hand a larger box (46 cm wide × 27 cm deep × 12 cm high) covered in black felt was placed on top of the original box. A 3 cm ridge with a tape measure attached ran along the side of the box that was only visible to the experimenter.

A life-sized adult right hand made of rubber was placed on top of the smaller box. The index finger on the rubber hand was placed at a distance of 10 cm from the index finger of the participant's hidden hand. The rubber hand was in full view of the participant and was covered from the wrist by a cape so that it was perceived as an extension of the participant's own right arm. A computer software package using Matlab provided a visual guide to control the speed of tactile stimulation. A timeline that depreciated at a speed of 3 cm/s (optimal for CT afferent stimulation) or 30 cm/s (non-optimal for CT afferent stimulation) was presented on a computer screen that only the experimenter could see. The duration of stimulation was 2 min and the absolute duration of stroking was kept constant across the four conditions such that 3cm/s trials consisted of 40 3 s strokes to the back/palm of the hand whilst 30 cm/s trials consisted of 120 1 s strokes to the back/palm of the hand. Thus, the actual time that the stroking finger was in contact with the participants' hands was the same in all four conditions.

### Outcome measures

#### Questionnaire ratings

To assess the strength of the illusion the following statements were adapted from the original Botvinick and Cohen (1998) questionnaire, which was presented to participants on one side of a sheet of A4 paper.

Q1: It felt as if I was feeling the stroking touch in the location where I saw the rubber hand touched.
Q2: It felt as if the rubber hand were my hand.
Q3: It seemed as though the touch I felt was caused by the touching on the rubber hand.
Q4: It felt as if my (real) hand was turning rubbery.
Q5: The rubber hand began to resemble my real hand in terms of shape, skin tone, freckles, or some other feature.

Three statements (Q1–Q3) were designed to capture different aspects of the illusory perception related to the sensation of touch on the rubber hand and the feeling of ownership of that hand. Statements Q4 and Q5 served as control questions for task compliance and susceptibility effects. The participants were asked to rate their level of agreement with the statements on a seven-point Likert scale ranging from “+3” (agree very strongly) to “−3” (disagree very strongly) where 0 corresponded to neither agreeing nor disagreeing. Botvinick and Cohen (1998) found that items 1, 2, and 3 had a significant tendency to evoke positive responses; therefore, we have used the sum of responses to items 1, 2, and 3 as a composite score indicating the perceived strength of ownership over the rubber hand (see also Lewis and Lloyd, 2010).

#### Pleasantness rating

Pleasantness ratings were used to identify the degree to which participants felt that the touch they received was pleasant. Participants were asked to verbally report their strength of agreement with the statement “The touch I felt was pleasant” using the same 7-point Likert scale from +3 to −3 as described above.

#### Proprioceptive drift

The amount of proprioceptive drift (i.e., the amount in cm the person feels that their own index finger has shifted toward the position of the index finger of the rubber hand after the stroking stimulation) was measured before and after each condition. The experimenter slowly ran her finger across the ridge of the larger box and asked the participant to say “stop” when they believed the experimenter's finger was directly in line with the location of their own hidden index finger (the artificial hand could not be seen during this part of the procedure). Using the tape measure attached to the ridge of the box, (which could only be seen by the experimenter), the distance between the participants' perceived location of their hidden finger and the actual location of their hidden finger was recorded (i.e., amount of proprioceptive drift).

### Procedure

Participants were asked to remove all jewellery from their right hand to ensure that visual congruence between the artificial hand and hidden hand was as high as possible and to reduce interference with the illusion from any personal items. Once they were comfortable and the participant's hand placed out of sight beneath the small and large black boxes, the first pre-test measure of proprioceptive position sense was obtained. The experimenter ran her finger along the length of the ridge on top of the large black box and the participant told the experimenter to stop when her finger was directly over the location of the participant's hidden index finger. The experimenter then read off the position of the stopping point from the tape measure. The large black box was then removed and the rubber hand was placed on top of the smaller box under which the participant's hand was concealed. The participant was told the index finger on their right hand and the index finger on the artificial hand would be stroked. They were asked to look at the artificial hand while their finger was stroked and to try and keep their hands and fingers still at all times. The participant was unable to see their own hidden hand during the experiment.

The experimenter stroked the participant's index finger and the index finger on the rubber hand at a rate of 3 or 30 cm/s for a period of 2 min. Immediately following the stroking stimulation the pleasantness rating was taken. The participant was asked to keep their hands and fingers still and the experimenter held up a sheet of paper displaying the pleasantness statement. The participant then verbally reported how strongly they rated their agreement with the statement using the 7-point Likert scale. The experimenter then placed the larger box over the original box again to enable a post-test measure of proprioceptive drift to be obtained. The same procedure was followed as for the pre-test proprioceptive estimate of hand position. Finally, the participant was provided with a copy of the self-report questionnaire and asked to rate the strength of the illusion by providing a value for each statement. The participant then put their hand back into the apparatus and completed the remaining conditions in the same way.

### Data analysis

The data were analyzed using SPSS v19.0. Initially descriptive statistics were used to explore the data and the Shapiro-Wilks test of normality (for small samples) was used to assess whether the data was normally-distributed. As data obtained from the self-report questionnaire statements and pleasantness ratings violated the assumption of normality, Friedman's ANOVA (a non-parametric test for related data) was used to examine the variance across all four conditions. The data were further analyzed using Wilcoxon signed-rank tests to compare the separate factors of hand orientation and stroking speed. The proprioceptive drift data were evaluated using repeated measures ANOVA. Finally, multiple regression analysis was employed to identify whether the subjective strength of the illusion (as measured through the questionnaire items) could best be predicted by the measure of proprioceptive drift or pleasantness ratings and whether proprioceptive drift of the hand (an objective rating) could be predicted by pleasantness ratings.

## Results

### Strength of the RHI

Table 1 provides the mean scores (±1 s.e.m) for all questionnaire items across all four conditions. The first three statements (Q1, 2, and 3) indicate the subjective experience of the illusion, whilst questions 4 and 5 are used as controls for subject compliance. Individual Wilcoxon signed-rank tests with an adjusted alpha level of 0.0125 were used to investigate whether there was an effect of condition (fast and slow stimulation to the back of the hand vs. fast and slow stimulation to the palm of the hand) on illusion strength (a composite measure of the sum of questionnaire items 1, 2, and 3 vs. a composite measure of the sum of questionnaire items 4 and 5). The analysis confirmed that participants provided higher agreement ratings for the questionnaire items relating to the illusion compared to the control items across all conditions (all *p* values ≤0.001). Having established that the first three items capture the illusion experience we used these scores to investigate the effect of site of stimulation and stroking speed on the subjective experience of the illusion. The Friedman test was non-significant [χ^2^(3) = 4.492, *p* = 0.213], indicating that even though the highest illusion ratings were given for the slowest stroking speed (back of the hand, *M* = 5.4; palm of the hand, *M* = 5.5) and the lowest rating was given when the palm of the hand was stroked at the fastest rate (*M* = 4.2) the subjective strength of the illusion did not differ significantly between stimulation to the back or palm of the hand or stroking speed^1^.

**Table 1:** **Mean scores (±1 s.e.m) for all questionnaire items across all four conditions**.

	**Condition A: back of hand, 3 cm/s**	**Condition B: back of of hand, 30 cm/s**	**Condition C: palm of hand, 3 cm/s**	**Condition D: palm of hand, 30 cm/s**
Q1: It felt as if I was feeling the stroking touch in the location where I saw the rubber hand touched	2.3 (0.2)	2.0 (0.3)	2.3 (0.3)	2.1 (0.2)
Q2: It felt as if the rubber hand were my hand	1.8 (0.3)	1.4 (0.4)	1.7 (0.3)	1.1 (0.4)
Q3: It seemed as though the touch I felt was caused by the touching on the rubber hand	1.3 (0.4)	1.1 (0.4)	1.5 (0.4)	1.0 (0.4)
**SUM of Q1–3**	**5.4 (0.8)**	**4.5 (1.0)**	**5.5 (0.8)**	**4.2 (0.8)**
Q4: It felt as if my (real) hand was turning rubbery	1.1 (0.4)	0.3 (0.4)	1.1 (0.4)	0.8 (0.4)
Q5: The rubber hand began to resemble my real hand in terms of shape, skin tone, freckles, or some other feature	0.2 (0.3)	−0.2 (0.3)	0.0 (0.3)	−0.6 (0.3)
**SUM of Q4 and 5**	**1.3 (0.6)**	**0.1 (0.7)**	**1.1 (0.6)**	**0.2 (0.6)**

**Figure 1:**
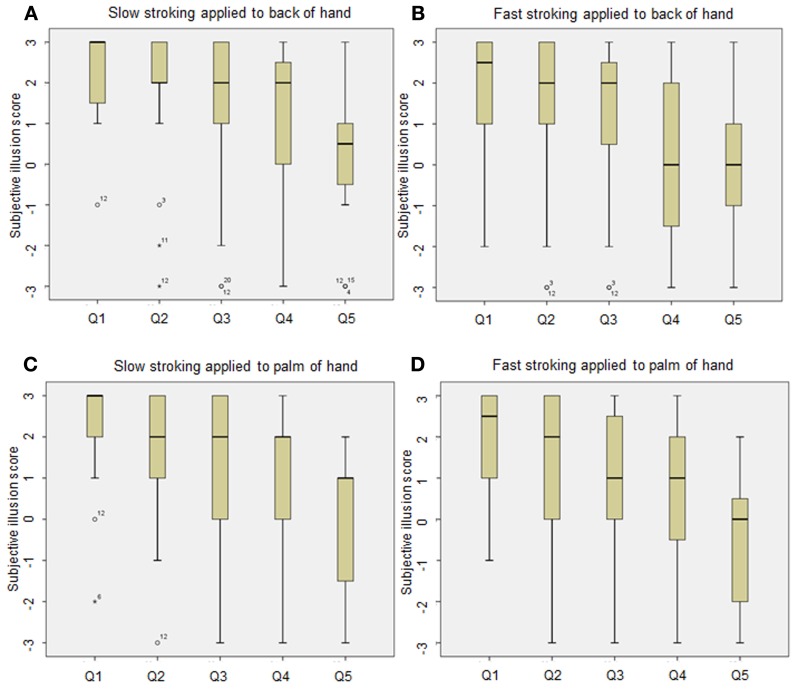
**Box plots displaying questionnaire responses for each condition** (**A**, slow stroking to back of hand; **B** fast stroking to back of hand; **C**, slow stroking to palm of hand and **D**, fast stroking to palm of hand): (Q1: It felt as if I was feeling the stroking touch in the location where I saw the rubber hand touched. Q2: It felt as if the rubber hand were my hand. Q3: It seemed as though the touch I felt was caused by the touching on the rubber hand. Q4: It felt as if my (real) hand was turning rubbery. Q5: The rubber hand began to resemble my real hand in terms of shape, skin tone, freckles, or some other feature). Participants indicated their response on a seven-point Likert scale ranging from “agree strongly” (+3) to “disagree strongly” (−3). *Intersecting lines* indicate the median response, bars indicate range and the box indicates inter-quartile range. Outliers are represented by O, extreme outliers are represented by *asterisks*.

Figure 1 shows box plots of the questionnaire data. As can be seen, the variability of the responses in the palm of the hand/fast stroking speed manipulation is far greater than the variability of the responses in the back of the hand/slow stroking speed manipulation. To further explore the results, the accumulated skew statistics were analyzed for each individual questionnaire statement and converted to Z-scores to assess significance. For the first questionnaire statement (“*It felt as if I was feeling the stroking touch in the location where I saw the rubber hand touched*”) all manipulations showed a statistically significant tendency to evoke positive responses (all *p*'*s* < 0.01) except for stimulation delivered to the palm of the hand at the fastest stroking rate (*p* > 0.05). Whilst all manipulations produced positive responses for questionnaire statement 2 (“*It felt as if the rubber hand were my hand*”), for questionnaire statement 3 (“*It seemed as if the touch I felt was caused by the touching on the rubber hand*”) only slow tactile stimulation applied to the back and palm of the hand produced positive responses (*p* < 0.05)^2^.

### Pleasantness ratings

Table 2 provides the mean scores (±1 s.e.m) for pleasantness ratings across all four conditions. The Friedman test revealed a significant effect of stroking type on pleasantness ratings [χ^2^(3) = 13.845, *p* = 0.003]. The Wilcoxon signed-rank test (with an adjusted alpha level of 0.0125) revealed that touch to the hand was rated significantly more pleasant overall when the speed of stroking was 3 vs. 30 cm/s (*M* = 1.5 vs. 0.8, respectively; *p* = 0.004). Pleasantness ratings were also higher when stimulation was applied to the back of the hand at the slower vs. faster stroking speed (*M* = 1.4 vs. 0.6, respectively; *p* = 0.006) with an approaching significant effect on the palm of the hand at the slower vs. faster stroking speed (*M* = 1.5 vs. 1.0; *p* = 0.018).

**Table 2:** **Mean scores (±1 s.e.m) for all the composite of questionnaire items 1, 2, and 3, proprioceptive drift scores (post-stroking measure—baseline measure in cm) and pleasantness scores across all four conditions**.

	**Condition A: back of hand, 3 cm/s**	**Condition B: back of of hand, 30 cm/s**	**Condition C: palm of hand, 3 cm/s**	**Condition D: palm of hand, 30 cm/s**
Composite questionnaire scores (range ±9)	5.4 (0.8)	4.5 (1.0)	5.5 (0.8)	4.2 (0.8)
Proprioceptive drift measures (cm)	4.3 (0.5)	4.7 (0.6)	3.1 (0.7)	3.0 (0.6)
Pleasantness scores (range ±3)	1.4 (0.2)	0.6 (0.3)	1.5 (0.2)	1.0 (0.2)

### Proprioceptive drift results

Table 2 provides the proprioceptive drift means (±1 s.e.m) across all four conditions. Repeated measures Two-Way within-subjects ANOVA with stimulation site (back of hand, palm of hand) and stroking speed (fast, slow) revealed a main effect of site of stimulation [*F*_(1, 23)_ = 5.317, *p* = 0.03], with greater drift measures for stimuli applied to the back vs. palm of the hand (*M* = 4.5 vs. 3.1 cm) but no main effect of stroking speed [*F*_(1, 23)_ = 0.121, *p* = 0.731] and no interaction between site of stimulation and stroking speed [*F*_(1, 23)_ = 0.530, *p* = 0.474].

### Correlations

Table 3 shows the correlations among questionnaire scores, pleasantness and proprioceptive drift across each of our four experimental conditions. Subjective illusion ratings (from questionnaire scores) was significantly positively correlated with proprioceptive drift in Condition A (slow touch to back of hand), B (fast touch to back of hand) and C (slow touch to palm of hand) but not Condition D (fast touch to palm of hand). Subjective illusion scores were also significantly related to pleasantness in Conditions A, B, and D. Proprioceptive drift was only significantly associated with pleasantness in Condition B.

**Table 3:** **Pearson product-moment correlation matrix among experimental conditions (A, slow stroking to back of hand; B, fast stroking to back of hand; C, slow stroking to palm of hand and D, fast stroking to palm of hand) and self-report measures of illusion strength (measured by response to the questionnaire statements), rated pleasantness of touch and proprioceptive drift (cm)**.

		**Questionnaire**	**Pleasantness**	**Drift**
Condition A	Questionnaire	–	0.434^*^	0.510^**^
	Pleasantness	0.434^*^	–	0.331^§^
	Drift	0.510^**^	0.331^§^	–
Condition B	Questionnaire	–	0.340^*^	0.486^**^
	Pleasantness	0.340^*^	–	0.386^*^
	Drift	0.486^**^	0.386^*^	–
Condition C	Questionnaire	–	0.085	0.370^*^
	Pleasantness	0.085	–	−0.009
	Drift	0.370^*^	−0.009	–
Condition D	Questionnaire	–	0.441^*^	0.240
	Pleasantness	0.441^*^	–	0.027
	Drift	0.240	0.027	–

* Denotes correlation is significant at the p < 0.05 level,

** denotes correlation is significant at the p < 0.01 level.

§ Correlation is significant at p = 0.057.

Multiple regression analysis was conducted to assess the contribution of pleasantness and proprioceptive drift to subjective illusion scores. None of the correlation coefficients presented in Table 3 were over 0.80 indicating that singularity was not an issue with these data. All predictors used in the regression analysis had Variance Inflation Factors that ranged between 1.000 and 1.175 and tolerance levels that ranged between 0.851 and 1.000, indicating that the data were not affected by multicolinearity (Miles and Shevlin, 2001).

### Prediction of subjective illusion scores

Multiple linear regression analysis was carried out to determine the relative contributions of proprioceptive drift and pleasantness ratings to subjective illusion scores. The results of this analysis are displayed in Table 4. For Conditions A, B, and D both proprioceptive drift and pleasantness contributed significantly to the prediction of subjective illusion strength. Examination of beta weights for the regression model revealed that proprioceptive drift was associated with higher subjective illusion scores in Condition A (β = 0.412, *p* < 0.05) and Condition B (β = 0.417, *p* < 0.05), whereas in Condition D pleasantness was significantly associated with higher illusion scores (β = 0.288, *p* < 0.05).

**Table 4:** **Regression statistics for all four experimental conditions with the dependent variable subjective scores on the questionnaire and independent variables pleasantness and drift**.

		**AdjR^2^ (%)**	***F***	***B***	***SE B***	**β**	***t***	**Individual *R*^2^ (%)**
A	Pleasantness	27.6	5.382^*^	1.019	0.645	0.297	1.581	7.9
	Drift			0.645	0.295	0.412	2.188^*^	15.1
B	Pleasantness	19.3	3.756^*^	0.630	0.713	0.179	0.884	2.8
	Drift			0.659	0.321	0.417	2.052^*^	14.7
C	Pleasantness	6.4	1.780	0.408	0.933	0.088	0.437	0.8
	Drift			0.449	0.244	0.371	1.840	13.8
D	Pleasantness	17.4	3.428^*^	1.545	0.674	0.435	2.292^*^	18.8
	Drift			0.324	0.269	0.288	1.203	5.2

Condition A, slow stroking to back of hand; Condition B, fast stroking to back of hand; Condition C, slow stroking to palm of hand and Condition D, fast stroking to palm of hand.

* Denotes significance at p < 0.05; AdjR ^2^, Adjusted R Square.

### Prediction of proprioceptive drift

To determine whether pleasantness also contributed to the magnitude of a more objective measure of illusion strength, proprioceptive drift, we performed an additional regression analysis with proprioceptive drift as the dependent variable. Examination of the beta weights for the regression model revealed that pleasantness contributed to the prediction of proprioceptive drift with an approaching significant effect in Condition B only (β = 0.386, *p* = 0.062). There were no other significant contributions.

In summary, although there were no main effects of either stroking site or stroking speed on questionnaire responses the analysis of response distributions revealed that there was a greater tendency among participants to give positive responses to the questionnaire items when the hand was stroked slowly to both the back and palm of the hand. It was also the case that slow stroking was rated as more pleasant than fast stroking. Slow stroking, then, produces higher subjective ratings both of illusion strength and pleasantness of touch. There was, however, no effect of stroking speed on the objective measure of illusion strength, proprioceptive drift. For this measure it was stroking site rather than stroking speed that affected performance, with greater proprioceptive drift being associated with stroking of the back of the hand rather than the palm. Furthermore, regression analyses showed that when stimulation was applied to the back of the hand, whether it was slow or fast, proprioceptive drift was the strongest predictor of subjective illusion strength. There was, nevertheless, a correlation between pleasantness ratings and illusion strength which indicated that drift was only the strongest predictor rather than being the unique predictor. When, however, fast stimulation was applied to the palm of the hand, pleasantness of stimulation uniquely predicted the strength of the illusion. Pleasantness was not, however, a significant predictor of proprioceptive drift in any of the four conditions.

## Discussion

The results of our study indicate that emotional feelings, particularly those related to pleasant touch to the body, are a key moderator of subjective body ownership. A recent study found that the RHI seemed to be stronger with slow stroking than with fast stroking as manifested in the difference in subjective embodiment between synchronous and asynchronous stroking of a rubber hand (Crucianelli et al., 2013). The present study also found that the subjective strength of the RHI was stronger with slow stroking than with fast stroking, as manifested by the tendency to given positive responses to questionnaire items that tapped the experience of embodiment. The present findings are also consistent with those of Crucianelli et al. (2013) in that they did not find any effect of stroking speed on proprioceptive drift, an objective measure of RHI strength. These authors concluded that the perceived pleasantness of affective touch may underlie subjective aspects of the RHI, such as the phenomenological sense of embodying the rubber hand, but that the more emotionally neutral fast stroking may be associated with the objective aspects of the illusion, such as limb position sense.

Crucianelli et al. suggest that their finding of the superiority of slow stimulation over fast in mediating the subjective aspects of the RHI should be replicated on hairy skin, but not on non-hairy skin, because of the important role of CT afferents in mediating affective touch. The present study, however, found that the picture seems to be somewhat more complex than this. Our results suggest an indirect role for affective touch on the subjective sense of embodiment. Moreover, the effect of slow stroking does not seem to be confined to stimulation to the back of the hand. It therefore seems doubtful as to whether the role of pleasantness in the RHI is solely underpinned by CT afferent activation.

It should be noted that pleasant touch is not uniquely associated with CT afferents: although CT afferents are not found in the palm of the hand, touch to glabrous skin is often felt as pleasant (Löken et al., 2011). A recent study by McGlone et al. (2012) showed that stroking the hairy skin of the forearm activated the posterior insula and orbitofrontal cortex to a greater extent than did stroking the non-hairy skin of the palm of the hand whereas stroking the palm of the hand activated somatosensory cortex to a greater extent than did stroking the forearm. These different patterns of neural activity were found even though both stroking sites were associated with the same psychophysical measures of intensity and pleasantness ratings. The posterior insula is a key brain region involved in bodily awareness (Karnath et al., 2005), body ownership and agency (Tsakiris et al., 2007b), and the subjective awareness and affective processing of bodily signals (Craig, 2002; Morrison et al., 2011). The mid-anterior OFC receives input from posterior insula and encodes subjective pleasure (Kringelbach and Rolls, 2004). A touch questionnaire administered by McGlone et al. revealed that emotional descriptors of the tactile sensation were higher for forearm stimulation whereas sensory descriptors were higher for palm stimulation. This means that, even though slow touch to the palm of the hand is experienced as being just as pleasant as slow touch to the hairy skin of the forearm, the feelings of pleasantness are underpinned by different psychological factors. The authors proposed that pleasant touch from hairy skin, which activates CT afferents and is processed in limbic-related cortex, represents innate (i.e., non-learned) touch whereas pleasant touch from glabrous skin, mediated by Aβ afferents, is processed in somatosensory cortex and represents a *learned* process dependent on previously remembered pleasant tactile experience. A recent developmental study in infants (2–16 months) also supports a role for anterior frontal reward-related regions in processing pleasant touch (Kida and Shinohara, 2013). These authors propose that gentle touch is important for normal mental development. We would argue this is because pleasant, gentle touch may be integral to the development of bodily self-awareness.

Pleasantness of touch, then, may result from tactile stimulation of either hairy or glabrous skin and, though this pleasantness may be mediated by different neural mechanisms, it is nevertheless associated with increased subjective RHI strength. Although we found an association between pleasantness and subjective illusion strength in the present study, it should be noted that when the back of the hand was stimulated, proprioceptive drift proved to be a stronger predictor of subjective illusion strength than perceived pleasantness. This finding is incompatible with the suggestion that subjective illusion strength is uniquely due to the affective aspects of touch associated with activation of CT afferents. A neat dissociation between subjective affective touch and objective limb position sense (as manifested in proprioceptive drift) is too simplistic. The factors that drive the RHI are more closely intertwined with one another than this picture would suggest and, as the regression analyses in the present study indicate, the main predictors of the subjective sense of embodiment may depend upon the type of stimulation received and the site at which it is received: proprioceptive drift was the strongest predictor of subjective illusion strength when stimulation was to the back of the hand, but pleasantness was the unique predictor of subjective illusion strength when the palm of the hand was stroked rapidly.

The fact that pleasantness was the strongest predictor of subjective illusion strength when CT afferents were *not* being stimulated highlights the complex interplay between the different perceptual inputs (affective touch and emotionally neutral spatial information) that underlie the RHI. In the case of the strong predictive role of pleasantness, when the palm was stroked rapidly this may have been a side-effect of superior spatial awareness of hand position, which was manifested in smaller proprioceptive drift in this condition. Reduced proprioceptive drift may have allowed the other factor, affective touch, to come through as the main driver of subjective feeling simply because position sense was too accurate to contribute greatly to the overall experience of the illusion.

The finding of smaller proprioceptive drift, i.e., more accurate hand position sense, in the palm stimulation condition is consistent with recent findings that demonstrate enhanced spatial attention to stimuli located on or near the palm of the hand when compared to stimuli located on or near the back of the hand. Brown et al. (2009) found that participants were more accurate when they made pointing movements to visual targets projected onto the palm of the hand than they were making pointing movements to targets projected onto the back of the hand. They suggest that this finding may be due to the fact that the palm of the hand contains more bimodal receptors that encode both visual and tactile stimuli. The greater spatial accuracy associated with both visual and tactile stimulation on the palm of the hand may account for the fact that smaller measures of proprioceptive drift were obtained in the present study with stimulation to the palm of the hand rather than to the back of the hand. Proprioceptive drift is, after all, a type of error in which the participant feels that his or her hand is located in a position different to its real position. The greater number of visual-tactile bimodal neurons associated with the palm of the hand, and hence, the greater spatial resolution of stimulation on this part of the body, may account for the reduction in this error when the palm of the hand was stimulated.

This interpretation is also consistent with the findings of Reed et al. (2010) who, using a cueing paradigm, found that visual targets near the palm of hand were detected more quickly than those near the back of the hand, even though the distance between the hand and target was the same in both cases. Detection of targets near the palm of the hand was also superior to detection of targets near the forearm. Reed et al. concluded that this may be because greater attention is devoted to the grasping space around the palm of the hand than to other areas around the hand and arm and that this is driven by visual-tactile bimodal neurons.

Pleasantness, then, is one of the factors that predict the subjective strength of the RHI. Pleasantness, however, was not a significant predictor of proprioceptive drift scores. Pleasant touch therefore seems to focus attention on the subjective *feelings* of ownership of the rubber hand but is unlikely to mediate felt changes in hand location or agency. Nevertheless, as has been suggested above, the influence of felt pleasantness may be most apparent when the contribution of position sense to the overall experience of the illusion is minimized. A study by Lewis et al. (2013) using a version of the rubber hand paradigm where one of the fingers of the rubber hand has been removed, found that stroking the space where the missing finger was located changed the subjective experience of the hand such that compelling additional tactile sensations were felt in the person's own hand (including tingly, tense, numb, aching, cold and heavy) but this also did not influence proprioceptive drift scores. Rohde et al. (2011) have also recently shown that subjective ratings and proprioceptive drift partially dissociate in the RHI: whereas subjective ratings only increased in synchronous stroking conditions, proprioceptive drift occurred with both synchronous and asynchronous stroking (similar findings have been reported by Tsakiris and Haggard, 2005; Durgin et al., 2007; Crucianelli et al., 2013; Lewis et al., 2013). Repetitive TMS over inferior parietal lobe has been shown to attenuate the strength of the RHI as measured by proprioceptive drift but subjective self-reports of “feeling” of ownership remained unaffected (Kammers et al., 2009). A more recent study by Kammers et al. (2011) demonstrated a causal link between the RHI and changes in body temperature whereby cooling the hand increased the strength of the RHI whereas warming the hand produced a decrease in the illusion but this effect was only seen when measured with proprioceptive drift scores and not with subjective ratings of body ownership. Longo et al. (2008) and Lewis and Lloyd (2010) using qualitative methods have shown partly independent components of ownership and localization of the hand based on introspection and Holmes et al. (2006) showed that proprioceptive mis-localization could be observed in the absence of ownership. The accumulated evidence suggests that different mechanisms are responsible for perceptual drift and feelings of ownership but that they both contribute to the overall experience of the illusion in a complex way. The relevant mechanisms might be uncovered through evidence from neuroimaging studies.

The first study to investigate the neural correlates of the RHI was by Ehrsson et al. (2004). Using the subjective onset of the illusion and questionnaire scores as a marker of illusion experience they found correlated activity in the frontal operculum, specifically the ventral premotor cortex (vPM) and intraparietal sulcus. More recent studies have shown that this activity in vPM is temporally stable over a period of 6-months in people who subjectively report the illusion (Bekrater-Bodmann et al., 2012) and may be required for dynamic changes in feeling of limb ownership driven by multisensory integration based on connections with other areas. A study by Zeller et al. (2011) found that lesions, which damaged areas connected with vPM, resulted in a failure to experience the rubber hand illusion in a large population of stroke patients. Interestingly, this was not related to asomatognosia, a condition commonly found in stroke patients, where part or all of the body is perceived as feeling transformed, strange or alien. This might suggest that the mechanisms responsible for ownership of the rubber hand and disownership of the person's own hand also dissociate during the RHI. A region of ipsilateral (right) posterior insula has also been shown to correlate with the degree to which a rubber arm is incorporated into one's body representation during the RHI using perceptual drift as an online measure (Tsakiris et al., 2007a). Dorso-posterior insula cortex receives input from both un-myelinated and thinly myelinated fibers including thermoreceptors, nociceptors, and visceral afferents and has a role in interoception (Craig, 2002). Previous studies have shown how artificial limbs can evoke the same feelings as real limbs and provide objective neurophysiological evidence that the rubber hand is fully incorporated into the body using their threat paradigms (e.g., Armel and Ramachandran, 2003; Lloyd et al., 2006; Ehrsson et al., 2007). It is possible, therefore, that processes other than multisensory integration are involved in generating and maintaining awareness of bodily self, one of which might be emotional self-awareness.

Neuroanatomical and neurophysiological studies have shown that feeling and emotions are important in establishing body ownership and embodiment and that there are strong links between emotion, interoception and the bodily-self (Damasio, 1994). In our study, although pleasantness ratings of the tactile stimulation were sometimes able to predict subjective illusion scores, they were not able to predict changes in proprioceptive drift. This might suggest that the mechanisms of visuo-tactile-proprioceptive integration underlying proprioceptive drift are *partially* independent of the processes causing feelings of ownership. There may be two partly overlapping pathways meditating the subjective (i.e., “feelings” of body ownership) and objective (i.e., perceived limb position) aspects of bodily awareness (based on a model of somatosensory processing proposed by Dijkerman and de Haan, 2007). Nevertheless, this differentiation between affective interoceptive information and neutral spatial position sense is not completely cut-and-dried: under some circumstances proprioceptive drift is the main predictor of illusion strength whereas under other circumstances it is pleasantness that is the unique predictor. Moreover, the effect of affective touch on the illusion may sometimes be indirect rather than directly causal.

Based on the available neuroimaging results proprioceptive drift activates structures mainly in the inferior parietal lobe and then projects to the posterior insula and vPM. The insula is concerned with higher-order somatosensory processing of the body related to subjective awareness and processing of body signals and the pathway from posterior to anterior insula is related to the subjective awareness of the body and bodily emotions (i.e., how you “feel”). Structures mediating subjective aspects of bodily awareness activate the posterior and anterior insula, vPM, and OFC. Neuropsychological studies support this division by showing that lesions to anterior parietal cortex lead to tactile and proprioceptive impairments not higher-order body awareness deficits (Chen et al., 2003) and that the anterior parietal cortex codes the perceived rather than physical location of the body. In contrast, studies have shown that anosognosia for hemiplegia is seen when right posterior insula is damaged (Karnath et al., 2005) and somatoparaphrenic symptoms are seen after right posterior insula lesions leading to disturbed sensations of limb ownership (Cereda et al., 2002; Baier and Karnath, 2008). Therefore, it seems that the subjective experience of one's own body is driven by an emotional response to one's body and that interoceptive awareness modulates the online integration of the body percept in the RHI (possibly in the posterior insula), mediated by bottom-up multisensory inputs for perception but modulated by top-down affectively relevant stimulus context.

## Conclusions and future studies

In conclusion, this study has demonstrated a role for affective systems in the subjective but not objective aspects of body representation. The application of affective illusions could be used to understand psychosomatic disorders, such as medically unexplained symptoms (MUS), which have been hypothesized to result from a distortion in perception, whereby top-down factors influence the processes of body representation (Brown, 2004). A recent study by Miles et al. (2011) showed that self-reported unexplained symptoms were associated with altered experience of the RHI, whereby a low score on the Somatoform Dissociation Questionnaire (SDQ-20; Nijenhuis et al., 1996) predicted a stronger response to the RHI on both questionnaire and proprioceptive measures of the illusion whereas a high-SDQ score predicted greater responses to the Perceptual Aberrations Scale (Chapman et al., 1978), a measure of bodily distortions in daily life. Tsakiris et al. (2011) have similarly shown that low interoceptive sensitivity predicted increased illusion ownership, which was not due to changes in perceptual drift or autonomic state. Somatisation, which is emotional distress in the form of somatic complaints, is likely mediated by the insula, which is involved in the representation of bodily states that provide positive and negative biases to cognitive decision making. For example, Gündel et al. (2008) showed increased insula activity in patients with somatoform pain vs. controls during an experimental induction of pain despite having the same subjective pain ratings as healthy controls. Future neuroimaging studies could employ the use of resting state methodologies to investigate whether medial brain systems of the default mode network dominate affective body representation in the RHI as it is possible that medial brain systems, involved in self-monitoring and regulating the internal world, mediate the subjective aspects of the illusion such as ownership (Northoff and Bermpohl, 2004), whereas lateral brain systems, involved in perceiving and responding to the external world, mediate the objective aspects such as perceived limb position.

## Author contributions

All authors designed the experiment. Victoria Gillis and Elizabeth Lewis collected the data. Donna M. Lloyd and Victoria Gillis analyzed the data. Donna M. Lloyd wrote a first draft of the manuscript. All authors read and commented on the final submitted version of the manuscript.

### Conflict of interest statement

The authors declare that the research was conducted in the absence of any commercial or financial relationships that could be construed as a potential conflict of interest.

## Footnotes

^1^We subsequently re-analyzed the data removing 3 participants who had a composite score of <3 across conditions (i.e., they gave 0 score to at least one of the questions). Only one participant (#12) failed to experience the illusion across all conditions and participants #3 and #11 failed across two conditions. Removing these participants from the analysis improved the strength of the relationship between subjective illusion scores and condition but did not make this significant [χ^2^(3) = 5.255, *p* = 0.154].

^2^Re-analysing the data with the Friedman test for each question (1, 2, and 3) revealed the following relationship between condition and questionnaire item: Question 1 [ χ^2^(3) = 1.330, *p* = 0.722]; Question 2 [χ^2^(3) = 4.566, *p* = 0.206]; Question 3 [ χ^2^(3) = 6.669, *p* = 0.083].
